# Altered Sensory Code Drives Juvenile-to-Adult Behavioral Maturation in *Caenorhabditis elegans*

**DOI:** 10.1523/ENEURO.0175-16.2016

**Published:** 2017-01-05

**Authors:** Laura A. Hale, Eudoria S. Lee, Alexandros K. Pantazis, Nikos Chronis, Sreekanth H. Chalasani

**Affiliations:** 1 Molecular Neurobiology Laboratory, The Salk Institute for Biological Studies, La Jolla, CA 92037; 2 Department of Mechanical Engineering, University of Michigan, Ann Arbor, MI 48109

**Keywords:** behavior, chemotaxis, development, neural circuits, sensory code

## Abstract

Adults perform better than juveniles in food-seeking tasks. Using the nematode *Caenorhabditis elegans* to probe the neural mechanisms underlying behavioral maturation, we found that adults and juveniles require different combinations of sensory neurons to generate age-specific food-seeking behavior. We first show that adults and juveniles differ in their response to and preference for food-associated odors, and we analyze genetic mutants to map the neuronal circuits required for those behavioral responses. We developed a novel device to trap juveniles and record their neuronal activity. Activity measurements revealed that adult and juvenile AWA sensory neurons respond to the addition of diacetyl stimulus, whereas AWB, ASK, and AWC sensory neurons encode its removal specifically in adults. Further, we show that reducing neurotransmission from the additional AWB, ASK, and AWC sensory neurons transforms odor preferences from an adult to a juvenile-like state. We also show that AWB and ASK neurons drive behavioral changes exclusively in adults, providing more evidence that age-specific circuits drive age-specific behavior. Collectively, our results show that an odor-evoked sensory code is modified during the juvenile-to-adult transition in animal development to drive age-appropriate behavior. We suggest that this altered sensory code specifically enables adults to extract additional stimulus features and generate robust behavior.

## Significance Statement

How does mature behavior arise during development? We use the nematode, *Caenorhabditis elegans*, to probe this question at the level of sensory neural circuits. Interestingly, despite having the same sets of sensory neurons, juveniles are worse than adults at seeking food. We found that adults use more neurons than juveniles to drive food-seeking behavior. We confirm the requirement for additional sensory neurons in the adult using functional imaging. Further, we show that these additional neurons participate exclusively in adults, and that blocking neurotransmission from these neurons transforms adult odor preferences to a juvenile-like state. We speculate that adults acquire additional features for a given food stimulus that allows them to find food more efficiently.

## Introduction

In most animals, adults and juveniles have large differences in their behavioral repertoire. For example, adult humans process social information differently than their adolescent counterparts (Blakemore 2008). Although imaging and behavioral studies in humans have suggested that the adolescent brain is plastic (Fuhrmann et al., 2015), less is known about the mechanisms driving this plasticity. Animal models, particularly hamsters, have been used to show that while juveniles and adults both detect social cues, they differ in their behavioral responses to those cues (Romeo et al., 1998; Petrulis 2009; Schulz et al., 2009). Additional studies have shown that adults and juveniles use different brain regions to process social cues, suggesting that differences in regional processing might explain the behavioral differences (Bell et al., 2013). However, the neural mechanisms that drive the differences between adult and juvenile behaviors remain poorly understood.

Work in nonmodel mammalian systems has attempted to understand the role of developmental behavioral differences at the level of sensory encoding and perception (Sarro et al., 2011). In particular, juvenile gerbils (*Meriones unguiculatus*) exhibit neural sensitivity that is superior to their behavioral threshold, suggesting that the capacity for robust behavior exists but is not executed in immature animals (Sarro et al., 2011). Here, we establish the nematode *Caenorhabditis elegans* as a genetically tractable model for understanding the mechanisms driving behavioral maturation during development. *C. elegans* is an ideal model for decoding developmental plasticity: its cellular lineage (Sulston and Horvitz, 1977; Sulston et al., 1983) and neuronal connectome (White et al., 1986) are fully mapped. Additionally, it undergoes a stereotyped developmental program that includes four juvenile larval stages (L1, L2, L3, and L4) separated by individual molts before reaching adulthood (Brenner, 1974). This rigorously executed developmental program provides well-defined stages that enable easier characterization of age-specific food-seeking behavior (Ward, 1973). In addition, developmental plasticity in the *C. elegans* system has been previously characterized. The *C. elegans* life cycle is regulated by environment; when juveniles, but not adults, are exposed to unfavorable conditions, they enter an alternate dauer diapause stage (Golden and Riddle, 1982, 1984). Furthermore, recent studies have shown that exposing *C. elegans* juveniles to stress affects both gene expression and behavior in adults (Hall et al., 2010; Sims et al., 2016). These results demonstrate that *C. elegans* exhibit age-specific behaviors that are subject to environmental influences and suggest that *C. elegans* is ideal for uncovering cellular-level mechanisms of behavioral maturation.

We focused our analysis on the maturation of food-seeking behavior, where adults are shown to have robust and quantitative readouts (Ward, 1973; Bargmann, 2006; Albrecht and Bargmann, 2011). Moreover, the sensory neurons driving these behaviors have also been mapped using cell ablations. The AWA chemosensory neurons are required for attraction to the food odor diacetyl (Bargmann et al., 1993; Larsch et al., 2015), while AWC neurons drive attraction to isoamyl alcohol, benzaldehyde, and 2,3-pentanedione (Bargmann et al., 1993; Wes and Bargmann, 2001, Bargmann, 2006). By contrast, AWB neurons drive repulsion from the volatile repellent, 2-nonanone (Troemel et al., 1997). When we examined food-seeking behavior in juveniles, we discovered that juveniles, compared with adults, have reduced attraction to the food-associated odor diacetyl and altered odor preferences. We show that both adults and juveniles detect diacetyl odor, but that adults use additional sensory neurons to encode diacetyl information. Further, we find that altering neurotransmission from the additional neurons can attenuate adult attraction to diacetyl and transform adults to a juvenile-like odor preference, linking changes in odor code to behavior. Our results highlight how the diacetyl odor code is altered during development and suggest that neurotransmitter pathways play a crucial role in generating plasticity in sensory neurons during maturation.

## Materials and Methods

### Odor chemotaxis

We conducted hatch-offs to obtain synchronously staged worms for behavioral analysis. Briefly, we placed 40–50 adults on 10-cm NGM agar plates seeded with an *Escherichia coli* OP50 lawn and allowed them to lay eggs for 2–3 h. The adults were removed, and egg-covered plates were incubated at 20°C for 48 h for L3 and 96 h for adult day 1 hermaphrodites.

Chemotaxis assays on square plates (Troemel et al., 1997) were conducted for 1 h at room temperature as previously described (Leinwand et al., 2015). Briefly, assay plates were poured using 11 ml of 1.6% agar solution containing 5 mm KPO_4_ (pH 6), 1 mm CaCl_2_, and 1 mm MgSO_4_. Animals were washed once in M9+MgSO_4_ followed by three washes in chemotaxis assay buffer (5 mm KPO_4_, 1 mm CaCl_2_, and 1 mm MgSO_4_). We used the following odors diluted in ethanol: (1) 2,3-butanedione (diacetyl; Sigma-Aldrich cat. # 11038), (2) benzaldehyde (Sigma-Aldrich cat. # 418099), (3) 2,3-pentanedione (Sigma-Aldrich cat. # 241962), (4) 2-nonanone (Sigma-Aldrich Cat. # 108731), and (5) pyrazine (Sigma-Aldrich cat. # P56003-10G) as indicated. Average chemotaxis index from six or more assays performed over at least three different days are shown.

Diacetyl preference assays were conducted as described by Fujiwara et al. (2016). Two-tailed, unpaired *t*-tests or one-way ANOVA were used to compare the responses of different genotypes or stages. Bonferroni correction for multiple comparisons was applied when appropriate. Source data and associated *p*-values for all behavior experiments are provided in Table 1.

**Table 1.** Raw data for chemotaxis and preference assays

Strain	Stage	Assay	Odor and concentration (%)	A + B or (total odor responders)	E + F or (total control responders)	A + B + C + D + E + F or (total responders)	CI or PI	SEM	Total plates (*n*)	Figure	*p*–value
N2	Adult	SPC	DAC 1	1548	71	1691	0.87	0.02	17	Fig. 1B*	—
N2	L3	SPC	DAC 1	1158	182	1641	0.59	0.03	19	Fig. 1B*	2.31E–08
N2	Adult	SPC	DAC 0.2	1383	77	1486	0.88	0.04	13	Fig. 1B	—
N2	L3	SPC	DAC 0.2	1325	335	1954	0.51	0.05	11	Fig. 1B	5.61E–06
N2	Adult	SPC	DAC 0.1	5901	638	6748	0.78	0.02	61	Fig. 1B*	—
N2	L3	SPC	DAC 0.1	4579	1318	7258	0.45	0.02	51	Fig. 1B*	4.03E–19
N2	Adult	SPC	DAC 0.05	1243	216	1505	0.68	0.04	14	Fig. 1B	—
N2	L3	SPC	DAC 0.05	688	368	1316	0.24	0.07	10	Fig. 1B	2.80E–05
N2	Adult	SPC	DAC 0.02	974	263	1294	0.55	0.08	12	Fig. 1B	—
N2	L3	SPC	DAC 0.02	982	647	2065	0.16	0.03	12	Fig. 1B	2.18E–04
N2	Adult	SPC	DAC 0.01	4144	1476	6040	0.44	0.03	51	Fig. 1B	—
N2	L3	SPC	DAC 0.01	3549	2315	7729	0.16	0.02	55	Fig. 1B	1.28E–12
N2	Adult	SPC	DAC 0.001	484	503	1197	–0.02	0.07	10	Fig. 1B	—
N2	L3	SPC	DAC 0.001	389	374	1064	0.01	0.05	11	Fig. 1B	0.51
N2	Adult	SPC	IAA 100	1117	16	1200	0.92	0.02	10	Fig. 1C	—
N2	L3	SPC	IAA 100	764	52	997	0.71	0.03	9	Fig. 1C	2.21E–05
N2	Adult	SPC	IAA 0.1	1784	97	1940	0.87	0.03	17	Fig. 1C	—
N2	L3	SPC	IAA 0.1	1111	119	1381	0.72	0.04	10	Fig. 1C	3.42E–03
N2	Adult	SPC	IAA 0.01	768	213	1012	0.55	0.10	9	Fig. 1C	—
N2	L3	SPC	IAA 0.01	776	485	1581	0.18	0.06	9	Fig. 1C	0.01
N2	Adult	SPC	BZ 0.1	1575	333	1947	0.64	0.04	17	Fig. 1D	—
N2	L3	SPC	BZ 0.1	1138	402	1843	0.40	0.06	15	Fig. 1D	5.32E–04
N2	Adult	SPC	BZ 0.01	1834	1000	3058	0.27	0.04	26	Fig. 1D	—
N2	L3	SPC	BZ 0.01	778	688	2164	0.04	0.03	17	Fig. 1D	3.73E–06
N2	Adult	SPC	PENT 0.1	838	10	862	0.96	0.02	7	Fig. 1E	—
N2	L3	SPC	PENT 0.1	903	34	1029	0.84	0.03	6	Fig. 1E	0.01
N2	Adult	SPC	PENT 0.01	633	66	720	0.79	0.04	6	Fig. 1E	—
N2	L3	SPC	PENT 0.01	1198	355	2023	0.42	0.08	8	Fig. 1E	3.28E–03
N2	Adult	SPC	NON 10	858	1516	2790	–0.24	0.06	23	Fig. 1F	—
N2	L3	SPC	NON 10	679	1601	2974	–0.31	0.05	20	Fig. 1F	0.26
N2	Adult	SPC	NON 2	433	492	1013	–0.06	0.06	10	Fig. 1F	—
N2	L3	SPC	NON 2	558	910	1946	–0.18	0.07	10	Fig. 1F	0.14
N2	Adult	SPC	NON 1	686	896	1738	–0.12	0.06	14	Fig. 1F	—
N2	L3	SPC	NON 1	530	785	1779	–0.14	0.05	12	Fig. 1F	0.80
N2	Adult	SPC	BZ 100	12	676	919	–0.72	0.04	9	Fig. 1*G*	—
N2	L3	SPC	BZ 100	8	782	1084	–0.71	0.04	10	Fig. 1*G*	0.81
N2	Adult	SPC	DAC 100	300	1177	1859	–0.47	0.05	19	Fig. 1H*	—
N2	L3	SPC	DAC 100	259	510	1120	–0.22	0.03	13	Fig. 1H*	3.21E–05
N2	Adult	Preference	DAC 0.1, PYR 0.8mg/ml	1222 (DAC)	761 (PYR)	1983	0.23	0.02	14	Fig. 1J*	—
N2	L3	Preference	DAC 0.1, PYR 0.8mg/ml	164 (DAC)	681 (PYR)	845	–0.61	0.04	12	Fig. 1J*	3.21E–12
N2	Adult	SPC	DAC 0.01	3826	1204	5418	0.48	0.02	46	Fig. 2A	—
AWA-	Adult	SPC	DAC 0.01	768	637	1541	0.09	0.07	12	Fig. 2A	<0.05
AWB-	Adult	SPC	DAC 0.01	953	492	1620	0.28	0.03	11	Fig. 2A	<0.05
AWC-	Adult	SPC	DAC 0.01	921	257	1290	0.51	0.05	11	Fig. 2A	>0.05
ASE-	Adult	SPC	DAC 0.01	1151	409	1614	0.46	0.08	12	Fig. 2A	>0.05
ASH-	Adult	SPC	DAC 0.01	750	276	1142	0.42	0.03	11	Fig. 2A	>0.05
ASK-	Adult	SPC	DAC 0.01	771	430	1276	0.27	0.05	10	Fig. 2A	<0.05
N2	L3	SPC	DAC 0.01	3253	1916	6859	0.19	0.02	48	Fig. 2A	—
AWA-	L3	SPC	DAC 0.01	340	393	997	–0.05	0.05	10	Fig. 2A	<0.05
AWB-	L3	SPC	DAC 0.01	812	470	1769	0.19	0.05	13	Fig. 2A	>0.05
AWC-	L3	SPC	DAC 0.01	874	495	1748	0.22	0.04	10	Fig. 2A	>0.05
ASE-	L3	SPC	DAC 0.01	887	421	1603	0.29	0.07	14	Fig. 2A	>0.05
ASH-	L3	SPC	DAC 0.01	582	401	1329	0.14	0.03	10	Fig. 2A	>0.05
ASK-	L3	SPC	DAC 0.01	494	391	1141	0.09	0.02	12	Fig. 2A	>0.05
N2	Adult	SPC	DAC 0.1	5901	638	6748	0.78	0.02	61	Fig. 2C*	—
AWA-	Adult	SPC	DAC 0.1	490	491	1096	0.00	0.04	10	Fig. 2*C*	<0.05
AWB-	Adult	SPC	DAC 0.1	949	85	1064	0.81	0.04	10	Fig. 2*C*	>0.05
AWC-	Adult	SPC	DAC 0.1	1391	112	1566	0.82	0.04	12	Fig. 2*C*	>0.05
ASE-	Adult	SPC	DAC 0.1	1079	125	1224	0.78	0.06	10	Fig. 2*C*	>0.05
ASH-	Adult	SPC	DAC 0.1	964	95	1108	0.78	0.05	10	Fig. 2*C*	>0.05
ASK-	Adult	SPC	DAC 0.1	909	242	1195	0.56	0.05	11	Fig. 2*C*	<0.05
N2	L3	SPC	DAC 0.1	4579	1318	7258	0.45	0.02	51	Fig. 2*C*	—
AWA-	L3	SPC	DAC 0.1	423	419	1191	0.00	0.03	10	Fig. 2*C*	<0.05
AWB-	L3	SPC	DAC 0.1	910	193	1428	0.50	0.03	13	Fig. 2*C*	>0.05
AWC-	L3	SPC	DAC 0.1	1223	247	1804	0.54	0.04	10	Fig. 2*C*	>0.05
ASE-	L3	SPC	DAC 0.1	1640	345	2278	0.57	0.03	16	Fig. 2*C*	<0.05
ASH-	L3	SPC	DAC 0.1	1099	227	1682	0.52	0.06	11	Fig. 2*C*	>0.05
ASK-	L3	SPC	DAC 0.1	668	253	1087	0.38	0.02	10	Fig. 2*C*	>0.05
N2	Adult	SPC	DAC 1	1548	71	1691	0.87	0.02	17	Fig. 2E*	—
AWA-	Adult	SPC	DAC 1	975	253	1369	0.53	0.07	12	Fig. 2*E*	<0.05
AWB-	Adult	SPC	DAC 1	1259	45	1417	0.86	0.01	12	Fig. 2*E*	>0.05
AWC-	Adult	SPC	DAC 1	642	58	792	0.74	0.05	10	Fig. 2*E*	>0.05
ASE-	Adult	SPC	DAC 1	893	22	931	0.94	0.02	10	Fig. 2*E*	>0.05
ASH-	Adult	SPC	DAC 1	805	24	866	0.90	0.02	10	Fig. 2*E*	>0.05
ASK-	Adult	SPC	DAC 1	808	115	953	0.73	0.06	12	Fig. 2*E*	>0.05
N2	L3	SPC	DAC 1	1158	182	1641	0.59	0.03	19	Fig. 2E*	—
AWA-	L3	SPC	DAC 1	1597	539	2611	0.41	0.04	15	Fig. 2*E*	<0.05
AWB-	L3	SPC	DAC 1	848	94	1130	0.67	0.03	10	Fig. 2*E*	>0.05
AWC-	L3	SPC	DAC 1	818	124	1256	0.55	0.03	10	Fig. 2*E*	>0.05
ASE-	L3	SPC	DAC 1	1131	72	1251	0.85	0.02	11	Fig. 2*E*	<0.05
ASH-	L3	SPC	DAC 1	715	47	920	0.73	0.03	11	Fig. 2*E*	>0.05
ASK-	L3	SPC	DAC 1	911	185	1364	0.53	0.03	12	Fig. 2*E*	>0.05
N2	Adult	SPC	DAC 100	300	1177	1859	–0.47	0.05	19	Fig.2-1B*	—
AWA-	Adult	SPC	DAC 100	239	758	1115	–0.47	0.11	10	Fig. 2-1*A*	>0.05
AWB-	Adult	SPC	DAC 100	187	625	1007	–0.43	0.07	11	Fig. 2-1*B*	>0.05
AWC-	Adult	SPC	DAC 100	115	893	1324	–0.59	0.05	14	Fig. 2-1*B*	>0.05
ASE-	Adult	SPC	DAC 100	301	660	1117	–0.32	0.07	10	Fig. 2-1*B*	>0.05
ASH-	Adult	SPC	DAC 100	320	272	751	0.06	0.10	10	Fig. 2-1*B*	<0.05
ASK-	Adult	SPC	DAC 100	90	703	852	–0.72	0.07	10	Fig. 2-1*B*	>0.05
N2	L3	SPC	DAC 100	259	510	1120	–0.22	0.03	13	Fig.2-1B*	—
AWA-	L3	SPC	DAC 100	666	242	1384	0.31	0.03	10	Fig. 2-1*A*	<0.05
AWB-	L3	SPC	DAC 100	280	381	994	–0.10	0.06	11	Fig. 2-1*A*	>0.05
AWC-	L3	SPC	DAC 100	248	477	1157	–0.20	0.04	10	Fig. 2-1*A*	>0.05
ASE-	L3	SPC	DAC 100	552	870	1797	–0.18	0.06	11	Fig. 2-1*A*	>0.05
ASH-	L3	SPC	DAC 100	286	166	655	0.18	0.07	10	Fig. 2-1*A*	<0.05
ASK-	L3	SPC	DAC 100	321	272	856	0.06	0.06	10	Fig. 2-1*A*	<0.05
N2	Adult	SPC	DAC 0.1	1758	163	2006	0.80	0.03	19	Fig. 4*B*	—
AWB::TeTx line 12	Adult	SPC	DAC 0.1	872	84	1003	0.79	0.04	11	Fig. 4*B*	0.4683
AWB::TeTx line 2	Adult	SPC	DAC 0.1	234	23	260	0.81	0.02	3	Table 1	0.9668
N2	Adult	SPC	DAC 0.01	1738	573	2955	0.39	0.03	25	Fig. 4*B*	—
AWB::TeTx line 12	Adult	SPC	DAC 0.01	634	214	949	0.44	0.07	10	Fig. 4*B*	0.8496
AWB::TeTx line 2	Adult	SPC	DAC 0.01	256	80	377	0.47	0.09	3	Table 1	0.8941
N2	Adult	SPC	DAC 0.1	855	79	990	0.78	0.03	10	Fig. 4*C*	—
AWC::TeTx	Adult	SPC	DAC 0.1	672	86	815	0.72	0.04	11	Fig. 4*C*	0.2740
N2	Adult	SPC	DAC 0.01	871	327	1385	0.39	0.04	10	Fig. 4*C*	—
AWC::TeTx	Adult	SPC	DAC 0.01	516	205	816	0.38	0.06	12	Fig. 4*C*	0.8106
N2	Adult	SPC	DAC 0.1	928	132	1128	0.71	0.03	11	Fig. 4*D*	—
ASK::TeTx	Adult	SPC	DAC 0.1	667	106	820	0.68	0.05	11	Fig. 4*D*	0.7824
N2	Adult	SPC	DAC 0.01	823	288	1229	0.44	0.03	11	Fig. 4*D*	—
ASK::TeTx	Adult	SPC	DAC 0.01	375	169	609	0.34	0.07	11	Fig. 4*D*	0.2431
N2	Adult	SPC	DAC 0.1	1988	207	2289	0.78	0.03	18	Fig. 4*E*	—
AWB,AWC,ASK::TeTx line 5	Adult	SPC	DAC 0.1	544	85	687	0.67	0.03	12	Fig. 4*E*	0.01149
AWB,AWC,ASK::TeTx line 2	Adult	SPC	DAC 0.1	662	147	898	0.57	0.06	11	Table1	0.01
N2	Adult	SPC	DAC 0.01	1044	261	1441	0.54	0.03	14	Fig. 4*E*	—
AWB,AWC,ASK::TeTx line 5	Adult	SPC	DAC 0.01	576	248	939	0.35	0.04	13	Fig. 4*E*	2.87E–03
AWB,AWC,ASK::TeTx line 2	Adult	SPC	DAC 0.01	476	215	827	0.32	0.06	11	Table1	6.34E–03
N2	Adult	Preference	DAC 0.1, PYR 0.8mg/ml	1222 (DAC)	761 (PYR)	1983	0.23	0.02	14	Fig. 4F*	—
AWB,AWC,ASK::TeTx line 5	Adult	Preference	DAC 0.1, PYR 0.8mg/ml	292 (DAC)	440 (PYR)	732	–0.20	0.03	10	Fig. 4*F*	6.62E–09
N2	L3	Preference	DAC 0.1, PYR 0.8mg/ml	164 (DAC)	681 (PYR)	845	–0.61	0.04	12	Fig. 4F*	—
AWB,AWC,ASK::TeTx line 5	L3	Preference	DAC 0.1, PYR 0.8mg/ml	47 (DAC)	145 (PYR)	192	–0.52	0.06	10	Fig. 4*F*	0.1926
N2	L3	SPC	DAC 0.1	719	290	1336	0.32	0.05	13	Fig. 4-1*A*	—
AWB::TeTx line 12	L3	SPC	DAC 0.1	1087	349	1835	0.40	0.05	11	Fig. 4-1*A*	0.3929
AWB::TeTx line 2	L3	SPC	DAC 0.1	675	240	1210	0.36	0.05	5	Table 1	0.9479
N2	L3	SPC	DAC 0.01	1410	752	2812	0.23	0.03	22	Fig.4-1A*	—
AWB::TeTx line 12	L3	SPC	DAC 0.01	470	231	956	0.25	0.04	11	Fig. 4-1*A*	0.8853
AWB::TeTx line 2	L3	SPC	DAC 0.01	71	42	166	0.17	0.16	3	Table 1	0.9606
N2	L3	SPC	DAC 0.1	602	260	1199	0.29	0.07	12	Fig.4-1B*	—
AWC::TeTx	L3	SPC	DAC 0.1	439	132	697	0.44	0.06	11	Fig. 4-1*B*	0.7025
N2	L3	SPC	DAC 0.01	503	427	1256	0.06	0.04	14	Fig. 4-1*B*	—
AWC::TeTx	L3	SPC	DAC 0.01	315	215	730	0.14	0.03	10	Fig. 4-1*B*	0.1650
N2	L3	SPC	DAC 0.1	617	256	1142	0.32	0.07	11	Fig. 4-1*C*	—
ASK::TeTx	L3	SPC	DAC 0.1	506	143	788	0.46	0.05	11	Fig. 4-1*C*	0.1666
N2	L3	SPC	DAC 0.01	435	357	1102	0.07	0.05	12	Fig. 4-1*C*	—
ASK::TeTx	L3	SPC	DAC 0.01	252	170	623	0.13	0.03	10	Fig. 4-1*C*	0.2759
N2	L3	SPC	DAC 0.1	900	251	1424	0.46	0.04	10	Fig. 4-1*D*	—
AWB,AWC,ASK::TeTx line 5	L3	SPC	DAC 0.1	375	89	622	0.46	0.08	10	Fig. 4-1*D*	0.6579
N2	L3	SPC	DAC 0.01	742	317	1373	0.31	0.06	10	Fig. 4-1*D*	—
AWB,AWC,ASK::TeTx line 5	L3	SPC	DAC 0.01	180	77	361	0.29	0.05	10	Fig. 4-1*D*	0.9266
N2	Adult	SPC	DAC 0.1	737	112	900	0.69	0.03	10	Fig. 5*B*	—
AWC::tom-1A	Adult	SPC	DAC 0.1	762	56	849	0.83	0.04	14	Fig. 5*B*	0.0221
N2	L3	SPC	DAC 0.1	624	231	1165	0.34	0.05	12	Fig. 5B*	—
AWC::tom-1a	L3	SPC	DAC 0.1	352	127	588	0.38	0.07	13	Fig. 5*B*	0.0192
N2	Adult	SPC	DAC 0.01	1525	519	2291	0.44	0.02	17	Fig. 5*C*	—
AWB::tom-1A line 18	Adult	SPC	DAC 0.01	598	133	841	0.55	0.03	10	Fig. 5*C*	0.0279
AWB::tom-1A line 10	Adult	SPC	DAC 0.01	771	174	1050	0.57	0.05	10	Table 1	0.0517
N2	L3	SPC	DAC 0.01	1410	752	2812	0.23	0.03	22	Fig. 5*C*	—
AWB::tom-1a line 18	L3	SPC	DAC 0.01	300	163	640	0.21	0.06	12	Fig. 5*C*	0.7991
AWB::tom-1a line 10	L3	SPC	DAC 0.01	141	64	280	0.28	0.12	6	Table 1	0.6714
N2	Adult	SPC	DAC 0.01	1875	633	2798	0.44	0.03	21	Fig. 5*D*	—
ASK::tom-1A line 1	Adult	SPC	DAC 0.01	612	129	802	0.6	0.04	12	Fig. 5*D*	3.20E–03
N2	L3	SPC	DAC 0.01	1410	752	2812	0.23	0.03	22	Fig. 5*D*	—
ASK:: tom-1a line 1	L3	SPC	DAC 0.01	244	103	467	0.30	0.10	10	Fig. 5*D*	0.8374
N2	Adult	SPC	DAC 0.01	813	264	1203	0.46	0.04	12	Fig. 5-1*A*	—
AWC::tom-1A	Adult	SPC	DAC 0.01	679	148	886	0.60	0.05	15	Fig. 5-1*A*	0.1612
N2	L3	SPC	DAC 0.01	362	357	974	0.01	0.04	10	Fig. 5-1*A*	—
AWC::tom-1a	L3	SPC	DAC 0.01	276	151	610	0.20	0.05	11	Fig. 5-1*A*	8.97E–03
N2	Adult	SPC	DAC 0.1	1179	102	1338	0.80	0.03	14	Fig. 5-1*B*	—
AWB::tom-1a line 18	Adult	SPC	DAC 0.1	544	20	575	0.91	0.04	10	Fig. 5-1*B*	0.0535
AWB::tom-1a line 10	Adult	SPC	DAC 0.1	645	41	711	0.85	0.06	11	Table 1	0.5762
N2	L3	SPC	DAC 0.1	1738	573	2955	0.39	0.03	25	Fig.5-1B*	—
AWB::tom-1a line 18	L3	SPC	DAC 0.1	212	105	406	0.26	0.06	10	Fig. 5-1*B*	0.1370
AWB::tom-1a line 10	L3	SPC	DAC 0.1	165	54	312	0.36	0.09	8	Table 1	0.9818
N2	Adult	SPC	DAC 0.1	1758	163	2006	0.80	0.03	19	Fig. 5-1*C*	
ASK::tom-1A line 1	Adult	SPC	DAC 0.1	621	52	684	0.83	0.04	12	Fig. 5-1*C*	0.9466
N2	L3	SPC	DAC 0.1	1738	573	2955	0.39	0.03	25	Fig.5-1C*	—
ASK:: tom-1a line 1	L3	SPC	DAC 0.1	242	72	397	0.43	0.08	10	Fig. 5-1*C*	0.5807
N2	Adult	SPC	BZ 0.1	1575	333	1947	0.64	0.04	17	Fig. 5-1*D*	—
AWC::tom-1A	Adult	SPC	BZ 0.1	706	181	998	0.53	0.05	10	Fig. 5-1*D*	0.0244
N2	L3	SPC	BZ 0.1	1138	402	1843	0.40	0.06	15	Fig. 5-1*D*	—
AWC::tom-1A	L3	SPC	BZ 0.1	375	148	683	0.33	0.04	10	Fig. 5-1*D*	0.4917
N2	Adult	SPC	IAA 0.1	1784	97	1940	0.87	0.03	17	Fig. 5-1*E*	—
AWC::tom-1A	Adult	SPC	IAA 0.1	602	60	706	0.77	0.04	10	Fig. 5-1*E*	0.1000
N2	L3	SPC	IAA 0.1	1111	119	1381	0.72	0.04	10	Fig. 5-1*E*	—
AWC::tom-1A	L3	SPC	IAA 0.1	536	76	701	0.66	0.05	10	Fig. 5-1*E*	0.2885
N2	Adult	SPC	NON 10	858	1516	2790	–0.24	0.06	23	Fig. 5-1*F*	—
AWC::tom-1A	Adult	SPC	NON 10	207	364	712	–0.22	0.06	8	Fig. 5-1*F*	0.9443
N2	L3	SPC	NON 10	679	1601	2974	–0.31	0.05	20	Fig. 5-1*F*	—
AWC::tom-1A	L3	SPC	NON 10	178	284	692	–0.15	0.11	8	Fig. 5-1*F*	0.3410

Summary counts for all chemotaxis behavior in response to varying odor concentrations. Chemotaxis index (CI) = [(#A + #B) – (#F + #E)]/(#A + #B + #C + #F + #E + #D). Diacetyl preference index (PI) = (total odor responders) – (total control responders)/total responders. *p*-values are also reported here. DAC, diacetyl; BZ, benzaldehyde; IAA, isoamyl alcohol; NON, 2-nonanone; PYR, pyrazine; SPC, square plate chemotaxis. *Wild–type data used in generating multiple figures.

### Tracking

To assess the speed of L3 and adults, we recorded their locomotion on modified chemotaxis assay plates using a Pixelink camera PL-741B and analyzed their movements using custom software (Calhoun et al., 2015). The modified chemotaxis assay is a variant of the odor preference assay described above (see also Fig. 1-1*B*). Briefly, animals were washed once in M9+MgSO_4_ followed by three times in S Basal. Ten to thirty animals were placed at the origin and allowed to move toward a diacetyl spot (1/1000) on a round 6-cm plate for 30 min. Data presented were collected from at least four plates over three different days.

### Calcium imaging

Transgenic animals expressing GCaMP family of calcium indicators under cell-selective promoters have been previously described (Leinwand et al., 2015). For this study, we recorded from sensory neurons expressing either GCaMP2.2b or GCaMP3. We developed a novel device to trap and record activity from neurons in L3 juveniles. Briefly (see also Fig. 3*A*), the L3 device contains channels of three thicknesses: the worm trap channel (10 μm thick), the stimulus-buffer flow channels (40 μm thick), and the inlet and outlets channel (65 μm thick; see also Multimedia File 1 to observe flow patterns and Multimedia File 2 for the AutoCAD design of the L3 device). Adult neurons were imaged as previously described (Chalasani et al., 2007; Chronis et al., 2007). Both adults and juveniles were exposed to identical diacetyl concentrations with stimulus added to the nose at *t* = 10 s and removed at *t* = 130 s in each recording. To determine odor responsiveness, we compared the averages and standard deviation of the ratio of Δ*F*/*F*_0_ for a given neuron stimulated with odor to buffer controls. In all imaging traces, the average fluorescence in a 3-s window for *t* = 3–6 s for on-responses (*F*_0ON_) and 121–124 s for off-responses (*F*_0OFF_) were used as baselines. Neurons whose Δ*F*/*F*_0_ value was >3 standard deviations above the buffer responses were counted as responders (see also Table 2).

**Table 2.** Sensory neurons that responded to the addition or removal of diacetyl

[DAC]	SN	Stage	+1 SD ON responder	+2 SD ON responder	+3 SD ON responder		+1 SD OFF responder	+2 SD OFF responder	+3 SD OFF responder
1 × 10^−7^	AWA	Adult	8	6	6	#	6	3	1
			0.67	0.50	0.50	%	0.50	0.25	0.08
1 × 10^−7^	AWA	L3	4	1	1		0	0	0
			0.29	0.07	0.07		0.00	0.00	0.00
1 × 10^−7^	AWB	Adult	1	1	0		5	5	5
			0.09	0.09	0.00		0.45	0.45	0.45
1 × 10^−7^	AWB	L3	0	0	0		3	1	0
			0.00	0.00	0.00		0.30	0.10	0.00
1 × 10^−7^	AWC	Adult	2	0	0		10	3	3
			0.18	0.00	0.00		0.91	0.27	0.27
1 × 10^−7^	AWC	L3	0	0	0		0	0	0
			0.00	0.00	0.00		0.00	0.00	0.00
1 × 10^−7^	ASE	Adult	1	0	0		1	1	1
			0.10	0.00	0.00		0.10	0.10	0.10
1 × 10^−7^	ASE	L3	0	0	0		2	2	1
			0.00	0.00	0.00		0.18	0.18	0.09
1 × 10^−7^	ASH	Adult	0	0	0		1	0	0
			0.00	0.00	0.00		0.07	0.00	0.00
1 × 10^−7^	ASH	L3	1	0	0		0	0	0
			0.09	0.00	0.00		0.00	0.00	0.00
1 × 10^−7^	ASK	Adult	1	0	0		5	3	1
			0.08	0.00	0.00		0.42	0.25	0.08
1 × 10^−7^	ASK	L3	4	0	0		1	0	0
			0.33	0.00	0.00		0.08	0.00	0.00
1 × 10^−4^	AWA	Adult	13	13	12		2	1	1
			1.00	1.00	0.92		0.15	0.08	0.08
1 × 10^−4^	AWA	L3	8	6	6		2	0	0
			0.50	0.38	0.38		0.13	0.00	0.00
1 × 10^−4^	AWB	Adult	2	2	0		9	9	8
			0.15	0.15	0.00		0.69	0.69	0.62
1 × 10^−4^	AWB	L3	0	0	0		0	0	0
			0	0	0		0	0	0
1 × 10^−4^	AWC	Adult	9	3	2		10	9	8
			0.53	0.18	0.12		0.59	0.53	0.47
1 × 10^−4^	AWC	L3	0	0	0		0	0	0
			0.00	0.00	0.00		0.00	0.00	0.00
1 × 10^−4^	ASE	Adult	3	0	0		0	0	0
			0.25	0	0		0.00	0.00	0.00
1 × 10^−4^	ASE	L3	1	0	0		4	3	2
			0.09	0.00	0.00		0.36	0.27	0.18
1 × 10^−4^	ASH	Adult	2	1	0		1	0	0
			0.20	0.10	0.00		0.10	0.00	0.00
1 × 10^−4^	ASH	L3	1	0	0		0	0	0
			0.08	0.00	0.00		0.00	0.00	0.00
1 × 10^−4^	ASK	Adult	0	0	0		0	0	0
			0.00	0.00	0.00		0.00	0.00	0.00
1 × 10^−4^	ASK	L3	0	0	0		2	1	1
			0.00	0.00	0.00		0.17	0.08	0.08

**Table 3.** Summary of all strains and genotypes

Strain	Genotype	Description	Figures
N2		Wild type	Figs. 1*B*–*H*,*J*; 1–1; 2*A*,*C*,*E*; 2–1*B*; 4*B*–*F*; 4–1; 5*B*–*D*; 5–1; Table 1
CX4	*odr-7 (ky4) X*	*“*AWA-*”*	Figs. 2A,*C*,*E*; 2–1*B*; Table 1
JN1715	*peIs1715 [str-1p::mCasp-1, unc-122p::venus]*	*“*AWB-*”*	Figs. 2A,*C*,*E*; 2–1*B*; Table 1
PY7502	*oyIs85 [ceh36delp::TU#813; ceh-36delp::TU#814; srtx-1p::GFP, unc-122p::dsRED]*	*“*AWC-*”*	Figs. 2A,*C*,*E*; 2–1*B*; Table 1
PR672	*che-1(p672) I*	*“*ASE-*”*	Figs. 2A,*C*,*E*; 2–1*B*; Table 1
JN1713	*peIs1713 [sra-6p::mCasp-1, unc-122p::mcherry]*	*“*ASH-*”*	Figs. 2A,*C*,*E*; 2–1*B*; Table 1
QS4 (PS6025)	*qrIs2 [sra-9p::mCasp-1, elt-2p::GFP]*	*“*ASK-*”*	Figs. 2A,*C*,*E*; 2–1*B*; Table 1
PY6554	*oyEx6554 [gpa-4p::GCaMP2.2b, unc-122p::dsRed]*	*“*AWA*”*	Figs. 3*C*–O, 3–1*A*–*D*; Table 2
PY7336	*oyEx7336 [str-1p::GCaMP3, unc-122p::dsRed]*	*“*AWB*”*	Figs. 3*C*–*O*; Table 2
CX10536	*kyEx2595 [str-2p::GCaMP2.2b;unc-122p::GFP]*	*“*AWC*”*	Figs. 3*C*–*O*; Table 2
IV388	*ueEx7 [gcy-7p::GCaMP3, unc-122p::GFP]*	*“*ASE*”*	Figs. 3*C*–*O*; Table 2
IV346	*kyEx2865 [sra-6p:: GCaMP3, unc-122p::GFP]*	*“*ASH*”*	Figs. 3*C*–*O*; 3–1*E*; Table 2
CX10981	*kyEx2866 [sra-9p::GCaMP2.2b; unc-122p::GFP]*	*“*ASK*”*	Figs. 3*C*–*O*; Table 2
IV746	*ueEx535 [str-1p::TeTx:sl2mcherry, elt-2p::GFP]*	*“*AWB::TeTx (line 2)*”*	Table 1
IV747	*ueEx536 [str-1p::TeTx:sl2mcherry, elt-2p::GFP]*	*“*AWB::TeTx (line 12)*”*	Figs. 4*B*; 4–1*A*; Table 1
IV216	*ueEx131 [odr3p:TeTx:sl2mcherry, elt-2p::GFP]*	*“*AWC::TeTx*”*	Figs. 4*C*; 4–1*B*; Table 1
CX11576	*kyEx3097 [sra-9p::tetx::sl2mcherry, elt-2p::GFP]*	*“*ASK::TeTx*”*	Figs. 4*D*; 4–1*C*; Table 1
IV683	*ueEx473 [str-1p::TeTx:sl2mcherry, odr-3p::TeTx:sl2mcherry, sra-9p::TeTx:sl2mcherry, elt-2p::GFP]*	*“*AWB,AWC, ASK::TeTx (line 2)*”*	Table 1
IV684	*ueEx474 [str-1p::TeTx:sl2mcherry, odr-3p::TeTx:sl2mcherry, sra-9p::TeTx:sl2mcherry, elt-2p::GFP]*	*“*AWB,AWC, ASK::TeTx (line 5)*”*	Figs. 4*E*; 4–1*D*; Table 1
PY10824 (IV387)	*pyEx10824 [ceh-36delp::tom-1A::sl2mcherry sense + anti-sense, unc-122p::GFP]*	*“*AWC::tom-1A*”*	Figs. 5*B*, 5–1*A*,*D*–F; Table 1
IV745	*ueEx531 [str-1p::tom-1:: sl2mcherry sense+anti-sense, elt-2p::GFP]*	*“*AWB::tom-1A (line 10)*”*	Table 1
IV742	*ueEx531 [str-1p::tom-1:: sl2mcherry sense+anti-sense, elt-2p::GFP]*	*“*AWB::tom-1A (line 18)*”*	Figs. 5*C*; 5–1*B*; Table 1
IV750	*ueEx539 [sra-9::tom-1A::sl2mcherry sense+anti-sense, elt-2p::GFP]*	*“*ASK::tom-1A (line 1)*”*	Figs. 5*D*; 5–1*C*; Table 1

Multimedia File 1. Movie showing the switching of the flow patterns between “stimulus on” and “stimulus off” in the novel L3-microfluidic device. A stimulus change occurs at 2 s in clip. Movie is sped up 30×. 10.1523/ENEURO.0175-16.2016.video.1

### Strains and molecular biology

Strains were cultured using standard practices (Brenner, 1974). A 4.5-kb sequence fragment for *str-1* promoter (AWB; Harris et al., 2014) was synthesized (Genscript) and used to express tetanus toxin and *tom-1A* sense and antisense constructs to block and increase neurotransmission, respectively (Schiavo et al., 1992; Gracheva et al., 2007). To misexpress tetanus toxin in AWB, AWC, and ASK simultaneously, we performed germline transformations by microinjection of plasmids (Mello and Fire, 1995) at 100 ng/μl each of *str-1p::TeTx:sl2mcherry*, *odr-3p:TeTx:sl2mcherry* (Calhoun et al., 2015), and *sra-9p::TeTx:sl2mcherry* (Calhoun et al., 2015) along with 10 ng/μl *elt-2p::gfp* as a co-injection marker. Similarly, to misexpress tetanus toxin in AWB, we injected *str-1p::TeTx:sl2mcherry* plasmid at 100 ng/μl and *elt-2p::GFP* plasmid at 10 ng/μl. We also generated lines specifically expressing *tom-1A* sense and antisense in AWB by injecting *str-1p::tom-1A* sense and anti-sense plasmid at 50 ng/μl and *elt-2p::GFP* plasmid at 10 ng/μl and in ASK by injecting *sra-9p::tom-1A* sense and anti-sense plasmid at 100 ng/μl and *elt-2p::GFP* plasmid at 10 ng/μl.

## Results

### Juveniles perform worse than adults at attractive chemotaxis behaviors

To test whether juveniles and adults perform similarly on odor-evoked food-seeking tasks, we used a chemotaxis assay in which animals can move toward or away from a volatile test compound (Fig. 1*A*). Worms were washed from growth plates and placed in a central region of the assay plates (termed “origin”; see also Materials and Methods). We found that >70% of animals in the third larval stage, L3, left the origin, allowing them to respond to the odor gradients. In contrast, <50% of the younger L2 and L1 larvae left the origin, suggesting that the chemotaxis assay is unsuitable to analyze the behavior of very young worms (Fig. 1-1*A*). Thus, we focused our analysis on L3 juveniles, comparing L3 and adult behavior in response to a number of volatile food-associated odors. We found that L3 juveniles were significantly less attracted than adults to diacetyl (Fig. 1*B*), isoamyl alcohol (Fig. 1*C*), benzaldehyde (Fig. 1*D*), and 2,3-pentanedione (Fig. 1*E*). We also found that L3 juveniles and adults have different locomotion speeds (Fig. 1-1*B*, *C*). To eliminate the possibility that defective L3 behavior is simply a result of slower movement speed, we tested their performance to additional odors. Both L3 and adults similarly avoided the volatile repellent 2-nonanone (Fig. 1*F*), suggesting that L3 juveniles are indeed competent to generate adult-like chemotaxis responses. We found additional evidence of similarity between L3 juveniles and adults in their responses to repellents. Both L3 juveniles and adults avoided high concentrations of the volatile odor, benzaldehyde (Fig. 1*G*; Yoshida et al., 2012). However, we observed that L3 juveniles are less repelled than adults from high concentrations of diacetyl (Fig. 1*H*), suggesting that diacetyl circuits are specifically altered during the juvenile-to-adult transition.

**Figure 1.**
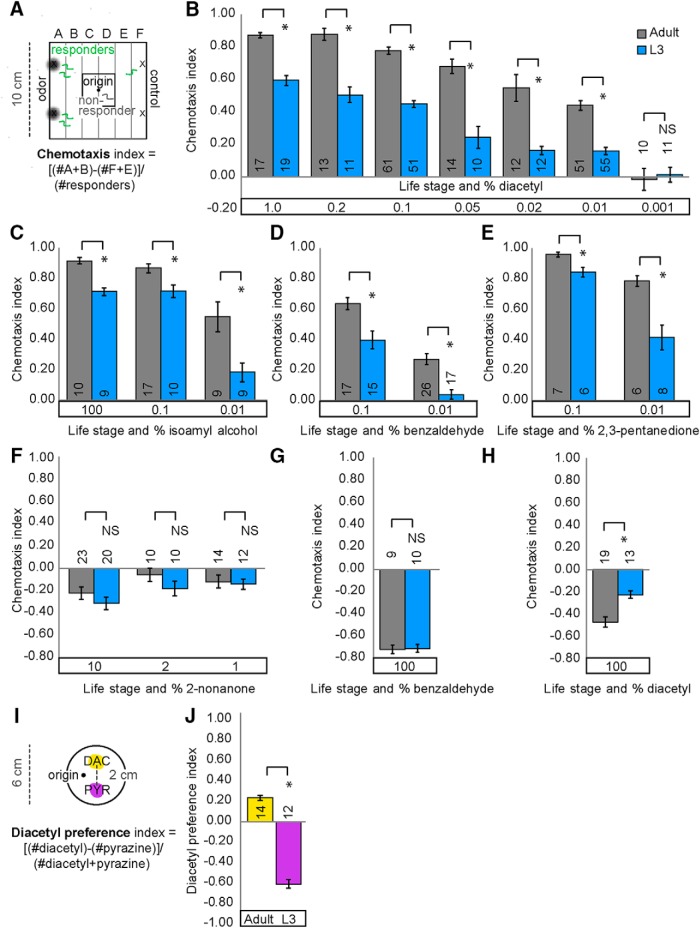
Juveniles and adults differ in attractive chemotaxis behavior. ***A***, Schematic of square plate (10 cm) assay used to assess chemotaxis behavior; both adult and L3 assays were conducted for 1 h (see also Materials and Methods). ***B***, Wild-type (N2) adult (gray) and L3 (blue) differ in their attraction across a range of attractive diacetyl concentrations. Wild-type adult and L3 also differ in chemotaxis behavior for various attractive AWC-sensed odors: isoamyl alcohol (***C***), benzaldehyde (***D***), and 2,3-pentandione (***E***). (F) Adults and juveniles are similarly repulsed by AWB-sensed 2-nonanone. Their behavior does not differ for repulsion to 100% benzaldehyde (***G***) but does for repulsive concentrations of diacetyl (***H***). ***I***, Schematic of round plate (6 cm) assay used to assess stage-specific choice preference (Fujiwara et al., 2016). ***J***, N2 adult prefer diacetyl (DAC) whereas N2 L3 prefer pyrazine (PYR). Averages and SEM are shown with numbers on each bar representing the number of assays. Brackets above bars indicate data compared using unpaired, two-tailed t-tests (*p < 0.05).

**Figure 1-1.**
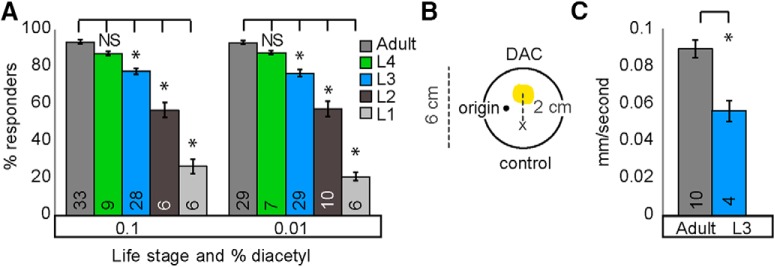
***A***, Larval worms generally respond less than adults in attractive diacetyl chemotaxis assays. Less than 50% of L1 and L2 stage worms responded in diacetyl behavioral assays so subsequent experiments focused on L3 and later stages. Averages and s.e.m. are shown. Numbers in brackets in or above each bar indicate number of assay plates. Data was compared using one-way ANOVA with Bonferroni correction for multiple comparisons (*=p<0.05). ***B***, Schematic of modified round plate assay used to track speed while 10-30 worms performed chemotaxis. (See also Materials and Methods). ***C***, Adult populations move faster than L3 populations during attractive diacetyl chemotaxis. Averages and s.e.m. are shown. Numbers in each bar indicate number of assay plates. Data were compared using unpaired, two-tailed *t*-tests (*p < 0.05).

To provide further evidence that L3 juveniles and adults respond differently to diacetyl, we conducted an odor preference assay in which animals were presented with opposing gradients of diacetyl and pyrazine (Fig. 1*I*; Fujiwara et al., 2016). We found that L3 juveniles prefer pyrazine, whereas adults prefer diacetyl (Fig. 1*J*), consistent with recently published results (Fujiwara et al., 2016). The chemotaxis results reveal that L3 juveniles have impaired responses to attractive and repulsive gradients of diacetyl and attractive gradients of isoamyl alcohol, benzaldehyde, and 2,3-pentanedione. Because AWA sensory neurons are required for diacetyl attraction and AWC neurons drive attraction toward isoamyl alcohol, benzaldehyde, and 2,3-pentandione (Bargmann et al., 1993; Bargmann, 2006), our results suggest that behaviors toward AWA- and AWC-sensed odors are altered during development. These results are consistent with a recent study showing that L3 juveniles, compared with adults, have reduced diacetyl attraction and altered odor preferences (Fujiwara et al., 2016). Moreover, these results show that *C. elegans* executes age-specific food and diacetyl-seeking behavior.

### Adults and juveniles require multiple sensory neurons to drive diacetyl chemotaxis

Because L3 juveniles have altered responses to both attractant and repellent concentrations of diacetyl, we focused our analysis on the mechanisms generating developmental plasticity to the AWA-sensed diacetyl. However, L3 juveniles, similar to adults, have all 12 pairs of amphid chemosensory neurons (Sulston and Horvitz, 1977; White et al., 1986). Thus, we tested the hypothesis that adults and juveniles differ in chemotaxis because they use, rather than possess, different sets of neurons to drive behavior. To probe the underlying sensory circuit generating attraction to diacetyl in adults and juveniles, we tested the behavioral responses of animals lacking individual chemosensory neurons including those that sense volatile odors: AWA, AWB, AWC, ASH (Larsch et al., 2013; Taniguchi et al., 2014; Zaslaver et al., 2015), water soluble chemicals: ASE (Suzuki et al., 2008), food: ASK (Calhoun et al., 2015), and temperature: AFD (Satterlee et al., 2001). We analyzed animals expressing caspase under ASH (*sra-6*), ASK (*sra-9*), AWC (*ceh-36*), and AWB (*str-1*) selective promoters (Beverly et al., 2011; Taniguchi et al., 2014) or mutants in transcription factors that specifically eliminate functional ASE (*che-1*), AFD (*ttx-1*), and AWA (*odr-7*) neurons (Fig. 2-1*A*; Sengupta et al., 1994; Satterlee et al., 2001; Uchida et al., 2003). Consistent with previous results (Bargmann et al., 1993), we found that adults missing AWA neurons were defective in their diacetyl attraction (Fig. 2*A*). Additionally, we found that adult animals lacking AWB and ASK neurons had reduced attraction to diacetyl (Fig. 2*A*). In contrast, we found that L3 animals lacking AWA, but not other chemosensory neurons, had severely impaired diacetyl attraction (Fig. 2*A*). These results indicate that whereas AWA drives attraction to diacetyl in both juveniles and adults, AWB and ASK are additionally required in adults (Fig. 2*B*).

**Figure 2.**
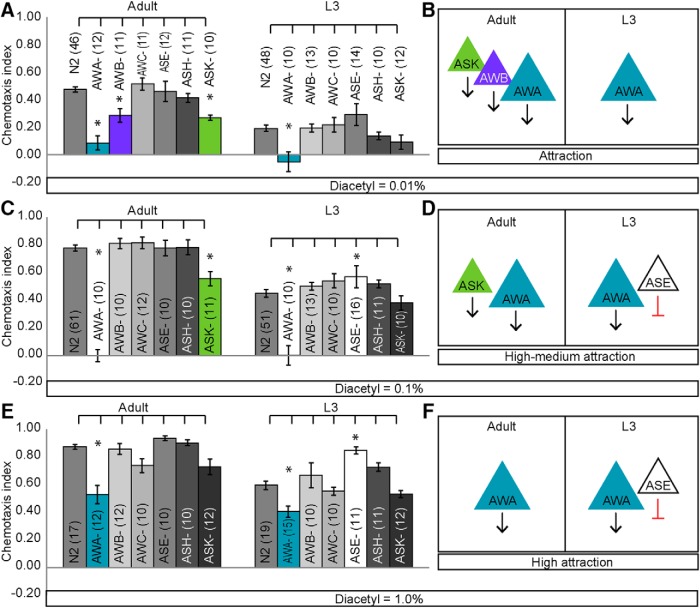
Juveniles and adults require different sets of sensory neurons for attractive diacetyl chemotaxis. ***A–E***, AWA is required in adults and juveniles for attraction to diacetyl. ***A***, ***B***, Interestingly, AWB and ASK are also required in adults, but not L3, for attraction. ***C***, ***D***, At intermediate levels of diacetyl, AWA and ASK are required in adults for attraction. ASE is also involved in L3 attraction. ***E***, ***F***, At a high level of diacetyl, only AWA is required in adults. By contrast, AWA and ASE play roles in L3 chemotaxis. Averages and SEM are shown in ***A***, ***C***, and ***E*** with numbers on each bar representing the number of assays. Data were compared using one-way ANOVA with Bonferroni correction for multiple comparisons (*p < 0.05). ***B***, ***D***, ***F***, Schematics of adult and L3 neurons required for attractive diacetyl behavior.

Odor sensory codes are modified by stimulus strength (Yoshida et al., 2012; Leinwand et al., 2015), so we tested whether the diacetyl sensory code is similarly altered by analyzing behavioral responses to other diacetyl concentrations. In each of the concentrations tested, we found that different combinations of sensory neurons are required in adults and juveniles. We found that at intermediate attractive concentrations, adults require AWA and ASK neurons, whereas juveniles use AWA and ASE sensory neurons for attractive chemotaxis (Fig. 2*C*, *D*). In contrast, at highly attractive concentrations, juveniles use ASE and AWA, whereas adults require only AWA to drive attraction (Fig. 2*E*, *F*). Next, we tested whether juveniles and adults use different sensory neurons to generate repulsion. We found that adults use ASH neurons, whereas juveniles use ASH, AWA, and ASK neurons to avoid undiluted diacetyl (Fig. 2-1). Together, these results indicate that adults and juveniles require different combinations of sensory neurons to drive behavioral responses to food-associated diacetyl odor.

**Figure 2-1.**
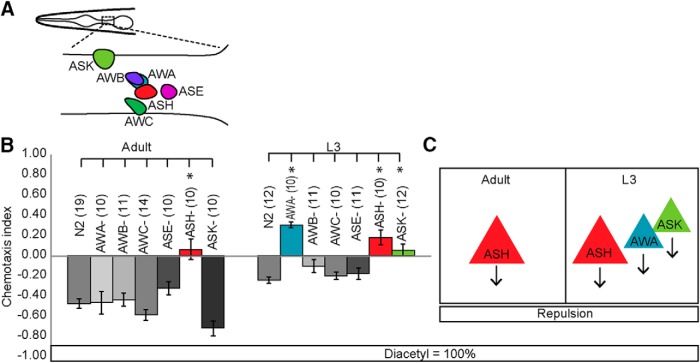
Juveniles and adults also have different sensory neurons for repulsive diacetyl behavior. ***A***, Schematic of sensory neurons tested in chemotaxis behavior. ***B***, Behavioral responses of adults and L3 to a repulsive concentration of diacetyl. ***C***, Schematic summarizing adult and L3 repulsive sensory neurons. Averages and SEM are shown. Numbers in brackets in or above each bar indicate number of assay plates. Data were compared using one-way ANOVA with Bonferroni correction (*p < 0.05).

### Juveniles and adults use different sets of sensory neurons to encode diacetyl

To test odor-evoked neuronal responses underlying odor behavior in juveniles, we developed a polydimethylsiloxane (PDMS)-based microfluidic device (Fig. 3*A*) for calcium imaging that successfully trapped L3 juveniles (Fig. 3*B*, *C*). We used a similar device to record adult sensory neuron responses to diacetyl (Fig. 3-1*A*, *B*; Chalasani et al., 2007; Leinwand et al., 2015) and validated our novel imaging device by recording activity from AWA neurons in L3 juveniles (Fig. 3-1*C*, *D*). Consistent with experiments using adults (Larsch et al., 2015), we found that juvenile AWA neurons also responded to addition of diacetyl odor (Fig. 3-1*A–D*) confirming the utility of the L3 trap to record juvenile neuronal responses.

**Figure 3.**
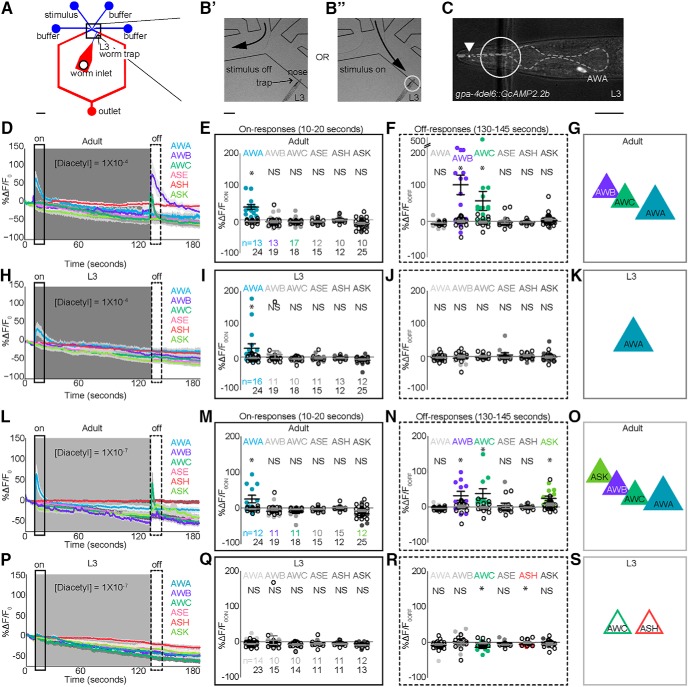
Juveniles and adults use different sensory neurons to encode diacetyl. ***A***, Schematic of novel PDMS-based microfluidic device for imaging L3 neurons (see also Materials and Methods). ***B***, Magnified views of L3 worm (circled region) trapped in device with stimulus off (***B′***) or stimulus on (***B″***). ***C***, Example L3 AWA expressing GCaMP2.2b under a reduced gpa-4 promoter. Anterior is to the left. White arrowhead indicates portion of dendritic tip in nose. Dashed gray outlines pharynx; used to landmark the position of sensory neuron cell bodies. ***D***, ***L***, Adult AWA, AWB, AWC, ASE, ASK, and ASH responses to different concentrations of diacetyl stimulus. Solid lines indicate average, and the light shadow represents SEM. Gray box here and in subsequent figures highlights stimulus duration (10–130 s). ***E***, ***M***, Scatterplots showing ratio of fluorescence changes (average) in a 10-s window (black rectangle) after stimulus addition (on-responses, 10–20 s). ***F***, ***N***, Average responses in a 15-s window (dashed rectangle) after stimulus removal (off-responses, 130–145 s). A similar analysis was performed on L3, and the resulting traces and scatter plots are shown in ***H–K*** and ***P–S***. Responders were estimated by comparing the average responses of individual neurons to the baseline controls (see Table 2 and Materials and Methods for details). Numbers of neurons imaged in each condition are shown in ***E***, ***I***, ***M***, and ***Q*** (*n*). Adult AWA neurons responded to the addition of all presented [diacetyl], whereas L3 AWA neurons responded only to the highest presented [diacetyl] (compare ***D***, ***H***, ***L***, and ***P***). Moreover, adult AWB, AWC, and ASK responded to the removal of odor. Data in ***E***, ***F***, ***I***, ***J***, ***M***, ***N***, ***Q***, and ***R*** were compared using two-tailed, unpaired *t*-tests (*p < 0.05). Solid colored circles represent responses of a given cell to odor, and open black circles represent the same cell’s response to a buffer-only control. Scale bar is 300 μm in A, 40 μm in ***B′***, and 10 μm in ***C***. ***G***, ***K***, ***O***, ***S***, Schematics of adult and L3 neurons encoding diacetyl.

**Figure 3-1.**
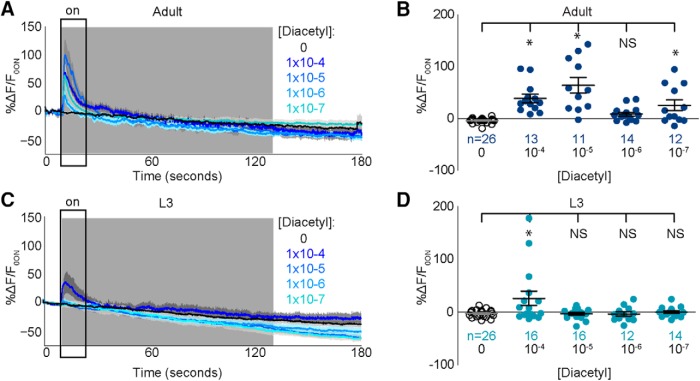
AWA encodes the addition of diacetyl in juveniles and adults. ***A***, Adult AWA responses to different diacetyl concentrations. Solid lines indicate average, and the light shadow represents SEM. Gray box here and in subsequent figures highlights stimulus duration (10–130 s). ***B***, Scatterplots showing average responses in a 10-s window immediately after stimulus addition (black rectangle). ***C***, ***D***, A similar analysis was performed on L3, and the resulting traces (***C***) and scatterplots (***D***) are shown. Solid colored circles represent responses of a given cell to odor, and open black circles represent the same cell’s response to a buffer-only control. Responders were estimated by comparing the average responses of individual neurons to the baseline controls (see Fig. 3-2 and Materials and Methods for details). Numbers of neurons imaged in each condition are shown in ***B*** and ***D*** (*n*). Adult AWA neurons responded to all presented [diacetyl], whereas L3 AWA neurons responded only to the highest presented [diacetyl] (compare ***B*** and ***D***). ***E***, Representative trace of L3 ASH responding to the addition of a repellent (black arrow indicates start of 2-nonanone stimulation). Data in ***B*** and ***D*** were compared using two-tailed, unpaired *t*-tests (*p < 0.05).

To confirm a functional role for the sensory neurons identified above through behavioral analysis, we examined their activity in L3 and adults. We hypothesized that diacetyl is encoded across multiple neurons, as previous studies have shown that chemical stimuli are encoded by the combined activity of multiple sensory neurons (Harris et al., 2014; Leinwand et al., 2015; Zaslaver et al., 2015). We examined the activity of several amphid sensory neurons, including those that sense volatile odors: AWA, AWB, AWC, ASH (Larsch et al., 2013; Taniguchi et al., 2014; Zaslaver et al., 2015); water-soluble chemicals: ASE (Suzuki et al., 2008); and food: ASK (Calhoun et al., 2015). Consistent with our behavioral analysis, we found that in both adults (Fig. 3*D–G*) and juveniles (Fig. 3*H–K*), AWA neurons respond to addition of diacetyl. Next, we analyzed the averaged responses of additional amphid sensory neurons. In both adults and L3, we found that, except for AWA, none of the neurons analyzed responded to the addition of diacetyl stimulus (Fig. 3*D*, *E*, *H*, and *I*). Rather, we observed that adult AWC and AWB neurons responded to the removal of stimulus (Fig. 3F), whereas we observed no such activation of these neurons in L3 juveniles (Fig. 3*J*). Although AWC was previously implicated in driving attraction to diacetyl (Chou et al., 2001), our results reveal novel roles for AWB and ASK in encoding diacetyl. Moreover, we found that adult AWA responded to the addition of a lower diacetyl concentration, whereas AWB, AWC, and ASK neurons were activated by its removal (Fig. 3*L–O*), suggesting that diacetyl strength is likely encoded by activity in different combination of sensory neurons. On average, L3 sensory neurons did not respond to a lower diacetyl concentration, but AWC and ASH were weakly suppressed (Fig. 3*P–S*). Collectively, the imaging results show that L3 and adults use different sets of chemosensory neurons to encode diacetyl. Adult AWA neurons encode the addition of diacetyl, and activity in AWB, AWC, and ASK encodes its removal, whereas L3 animals use AWA activity to encode diacetyl at a higher concentration (Fig. 3*G*, *K*, *O* and *S*). These results are consistent with recent studies confirming a role for AWC (Larsch et al., 2015; Fujiwara et al., 2016) along with AWA in driving diacetyl attraction.

We observed discrepancies in the sensory circuits identified using behavior or neural activity as readouts for neuronal function. Our behavior analysis showed that adults use ASK and AWB neurons in addition to AWA to drive attraction, whereas our imaging analysis also identified a role for AWC in encoding diacetyl in the adult. Consistent with previous observations in the benzaldehyde circuit (Leinwand et al., 2015), we found that different combinations of sensory neurons encode the concentration of diacetyl stimulus. We suggest that these discrepancies may arise from variations in the stimuli that the animals experience in the two assays: animals respond to a gradient of diacetyl stimulus (with the concentration of the point source indicated) in behavior assays, and imaging studies record neuronal responses to a known concentration of odor delivered to the nose of the animal.

### Altering the adult sensory code transforms adult behavior to juvenile-like states

We hypothesized that altering the adult sensory code, identified in behavioral and imaging experiments, by reducing neurotransmission from the additional sensory neurons that function specifically in adults for diacetyl-evoked behavior (Fig. 4*A*) would transform adult behavior into a L3-like state. We selectively blocked neurotransmission individually in AWB (Figs. 4*B* and 4-1*A*), AWC (Fig. 4*C* and 4-1*B*), and ASK (Figs. 4*D* and 4–1*C*) and in all three (Figs. 4*E* and 4-1*D*) by expressing tetanus toxin (TeTx). Previously, TeTx has been shown to block synaptic neurotransmission by cleaving synaptobrevin (Schiavo et al., 1992). In support of our hypothesis, TeTx misexpression in AWC, AWB, and ASK neurons together attenuates adult but not L3 diacetyl attraction (Figs. 4*E* and 4-1*D*). These results confirm our model that AWC, AWB, and ASK do not participate in driving diacetyl attraction in L3 juveniles. Moreover, blocking neurotransmission from AWC, AWB, and ASK individually did not affect adult (Fig. 4*B–D*) or L3 behavior (Fig. 4-1*A–C*), suggesting that these sensory neurons might be redundant in their ability to contribute to adult attraction to diacetyl. Additionally, we tested whether AWB, AWC, and ASK are required to generate age-appropriate odor preferences. We found that blocking neurotransmission from AWC, AWB, and ASK transformed adult preferences from diacetyl to pyrazine, without altering L3 odor preferences (Fig. 4*F*). These results suggest that neurotransmitter release from additional sensory neurons plays a crucial role in generating adult-specific responses: enhanced attraction to, and a preference for, diacetyl.

**Figure 4.**
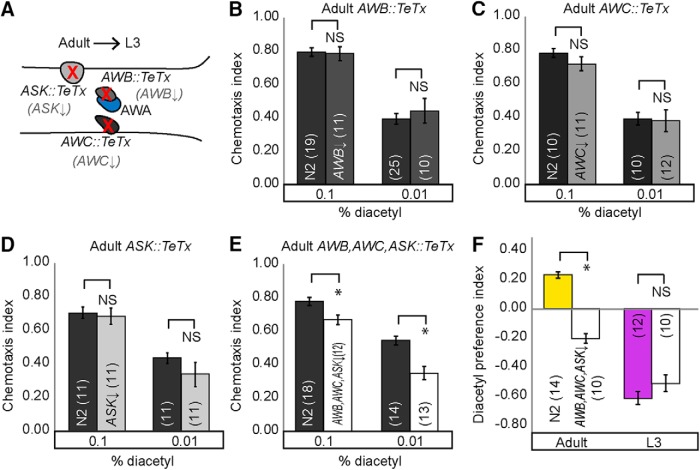
Knocking down neurotransmission of cells specific to adult circuit results in juvenile-like behavior. ***A***, Schematic for transforming adult to L3 behavior by misexpressing TeTx in AWB, AWC, and ASK neurons, and consequently decreasing synaptic transmission from those cells. Chemotaxis is not altered when TeTx is misexpressed individually in AWB*, AWC, or ASK (***B–D***), but does decline when misexpressed collectively in AWB, AWC, or ASK* (***E***). ***F***, Adults have a juvenile-like odor preference. Adult worms with decreased synaptic transmission in AWB, AWC, and ASK neurons prefer pyrazine, unlike their wild-type counterparts. Averages and SEM are shown with numbers on each bar representing the number of assays. Data were compared using unpaired, two-tailed *t*-tests (*p < 0.05) and, when appropriate, with Bonferroni correction for multiple comparisons (***B***, ***E***). *See also Table 1 for additional tested lines.

**Figure 4-1.**
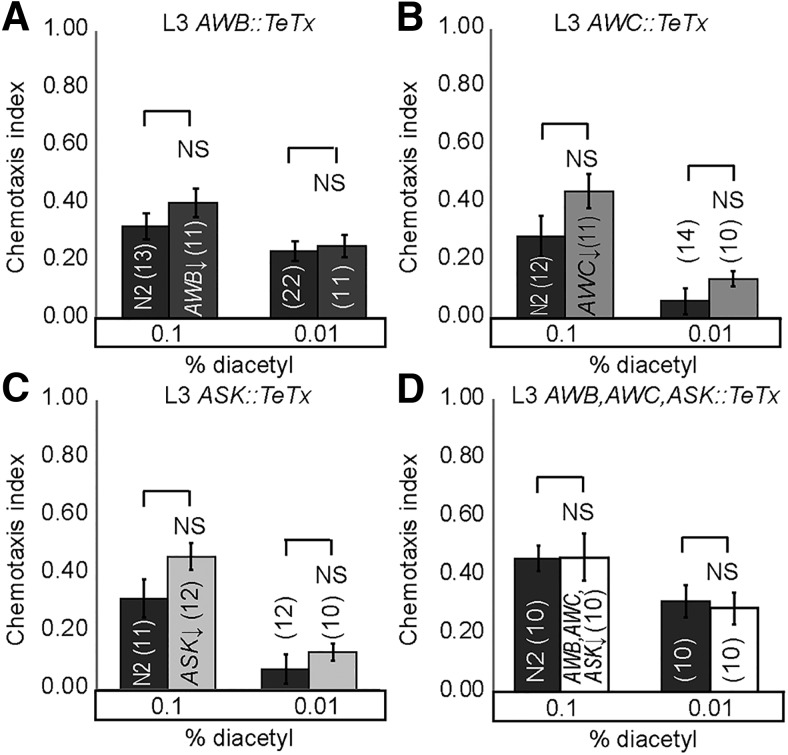
Manipulating AWB, AWC, and ASK sensory neuron activity in L3. Decreasing synaptic transmission by TeTx misexpression in L3 AWC (***A***), AWB* (***B***), ASK (***C***), or all three (***D***) does not alter chemotaxis. Averages and SEM are shown. Numbers in brackets in or above each bar indicate number of assay plates. Data were compared using two-tailed, unpaired *t*-tests (***B***) with Bonferroni correction, *p < 0.05. See also Table 1 for additionally tested lines.

To further manipulate the adult and juvenile sensory codes, we artificially increased neurotransmission from target sensory neurons. Knocking down *tomosyn* (*tom-1A* in *C. elegans*) increases both classic neurotransmission and neuropeptide signaling by upregulating dense-core vesicle release (Gracheva et al., 2007; Leinwand et al., 2015) and synaptic vesicles (Hu et al., 2013). We predicted that increasing neurotransmission from juvenile or adult sensory neurons detecting diacetyl would improve diacetyl attraction (Fig. 5*A*). Consistent with our hypothesis, we found that increasing neurotransmission from AWC improves diacetyl attraction in both juveniles and adults (Fig. 5*B*), confirming a role for this neuron at both life stages. This result is inconsistent with our calcium imaging experiments, in which we did not observe juvenile AWC neuronal activity in response to diacetyl. However, we suggest that calcium imaging might not reveal all AWC neural activity, as has been previously reported for other amphid sensory neurons (Zahratka et al., 2015). In contrast, we found that increasing neurotransmission from AWB and ASK improved diacetyl attraction in adults, but not juveniles (Figs. 5*C*, *D*, and 5-1). These data are consistent with our behavioral and imaging analysis and suggest that AWB and ASK are exclusively used in adults for diacetyl attraction.

**Figure 5.**
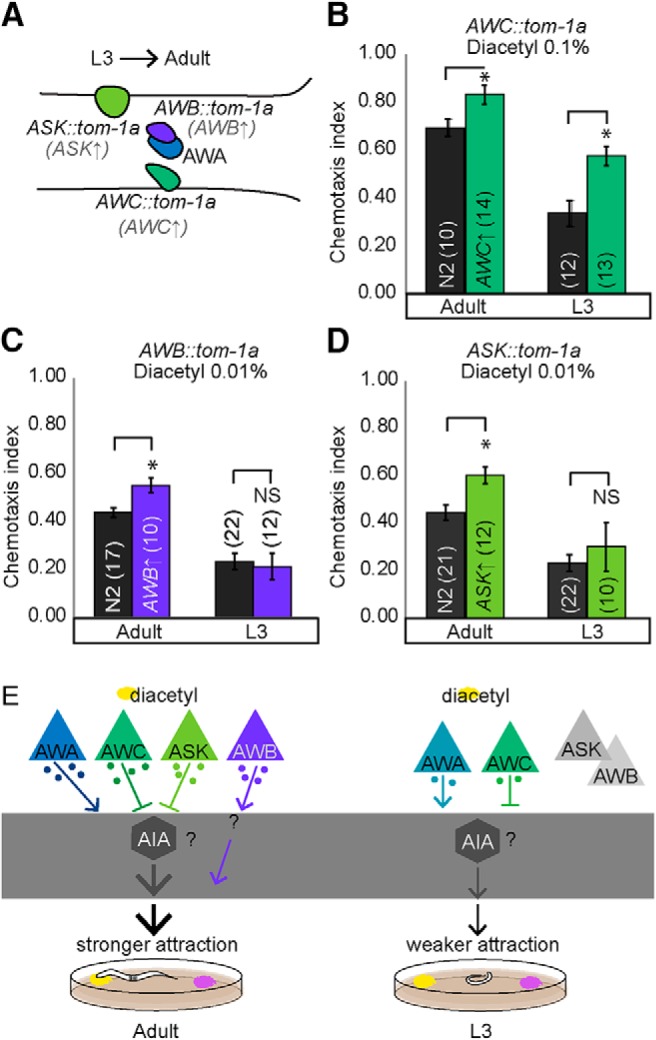
Increasing neurotransmission from cells specific to the adult circuit enhances diacetyl attraction in a contextually appropriate manner. ***A***, Schematic transforming L3 to adult behavior by knocking down *tom-1A* in AWC (***B***), AWB (***C***), and ASK (***D***). These manipulations specifically increase synaptic transmission from sensory neurons. ***B–D***, Enhanced attraction in adults is observed only for concentration-appropriate sensory circuits (see also Figs. 2 and 5-1*A–C*). Chemotaxis improves in L3 worms lacking tom-1A in AWC (***B***) but not for AWB and ASK tom-1A knockdowns (***C***, ***D***). Averages and SEM are shown in ***B–D*** with numbers on each bar representing the number of assays. Data were compared using unpaired, two-tailed *t*-tests (*p < 0.05). ***E***, Developmental differences in neurotransmission may underlie plasticity of diacetyl attraction.

**Figure 5-1.**
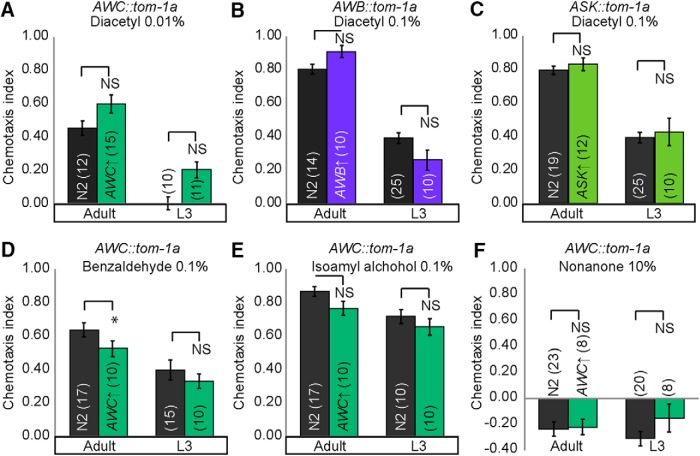
Increasing neurotransmission from AWB, AWC, and ASK sensory neurons in adults and L3. Increasing synaptic transmission by knocking down tom-1a in AWC (***A***), AWB (***B***), and ASK (***C***) does not enhance behavioral performance of adults and juveniles to low or higher concentrations of diacetyl. Tom-1A knockdown also does not enhance behavioral performance of adults and juveniles to benzaldehyde (***D***), isoamyl alcohol (***E***), or 2-nonanone (***F***). Averages and SEM are shown. Numbers in brackets in or above each bar indicate number of assay plates. Data were compared using two-tailed, unpaired *t*-tests, *p < 0.05.

## Discussion

Recent work showed that the diacetyl sensory circuit is modified by the developing germline leading to more robust attraction in the adult (Fujiwara et al., 2016). However, our results suggest a second nonexclusive mechanism that also explains developmental plasticity for diacetyl-evoked behavior.

In this study, we identify a novel role for altered sensory coding in driving the maturation of chemosensory circuits during development. Our imaging experiments reveal that adult AWA sensory neurons respond to the addition of diacetyl, whereas AWC, AWB, and ASK neurons respond to the removal of stimulus in a concentration-dependent manner. This combinatorial odor code has also been previously observed where multiple sensory neurons encode benzaldehyde (Leinwand et al., 2015) and isoamyl alcohol (Yoshida et al., 2012). Although AWC neurons have been implicated in diacetyl attraction (Chou et al., 2001), our results reveal a novel role for AWB and ASK in diacetyl attraction. We speculate that in adults AWB, AWC, and ASK neurons may initially be hyperpolarized by the addition of diacetyl and are activated upon its removal (Fig. 3*D*). Previous studies have identified a key role for AWA sensory neurons and the downstream AIA interneurons in sensing a broad range of diacetyl concentrations (Larsch et al., 2015). Additionally, this study also found that AWC sensory neurons acted upstream of these AIA interneurons in generating diacetyl attraction (Larsch et al., 2015). Previously, AWC neurons have also been shown to suppress AIA interneurons (Chalasani et al., 2010). Similarly, ASK might also inhibit AIA activity, as ablating ASK and AIA neurons has opposing effects on behavior (Gray et al., 2005). Because both AWC and ASK sensory neurons synapse onto AIA interneurons (White et al., 1986), we further hypothesize that these sensory neurons are likely to suppress AIA activity upon removal of diacetyl stimulus. Suppressing AIA interneurons increases turn behavior (Chalasani et al., 2007), perhaps enabling the animal to move toward the attractant. In addition, AWB neurons can function downstream of AWA neurons in refining attraction to benzaldehyde (Leinwand et al., 2015). Thus, we suggest that AWA neurons might also signal to AWB neurons to modify diacetyl attraction. Collectively, the combined activity across AWA, AWB, AWC, and ASK neurons enables adults to maintain strong diacetyl attraction, perhaps by modifying turn behaviors (Fig. 5*E*).

Through behavioral analyses of genetic mutants and transgenic animals, we show that both L3 and adults use AWA and AWC neurons to encode diacetyl, but that adults additionally use AWB and ASK neurons to encode the odor. By altering neurotransmission, we show that these additional sensory neurons play a crucial role in driving the enhanced adult attraction to diacetyl. Previous work has shown that neurotransmitter signaling can be altered by sensory context or aging (Leinwand and Chalasani, 2013; Leinwand et al., 2015). As development progresses, sensory experience, internal signals, and unfurling genetic programs modify the underlying neural pathways, perhaps at the level of neurotransmitter signaling, leading to a more complex and diverse adult-specific diacetyl sensory circuit that includes additional sensory neurons. We suggest that the larger adult circuit plays a crucial role in generating stronger attraction in the adults, perhaps by encoding more stimulus features or enabling a larger dynamic range for detection. Our results are consistent with those observed in desert locusts, in which adults show an increased number of chemical stimuli–responding sensory neurons compared with juveniles (Anton et al., 2002). We speculate that developmental plasticity allows juveniles to adapt to changes in their local environment, generating adults that can appropriately use available resources and even compensate for early negative experiences. This speculation is consistent with the observation that adult zebra finch males that experience starvation during development adapt compensatory mechanisms to enhance their reproductive fitness (Krause and Naguib, 2015).

## Synthesis

Reviewing Editor: Piali Sengupta, Brandeis University Decisions are customarily a result of the Reviewing Editor and the peer reviewers coming together and discussing their recommendations until a consensus is reached. When revisions are invited, a fact-based synthesis statement explaining their decision and outlining what is needed to prepare a revision will be listed below. The following reviewer(s) agreed to reveal their identity: Koutarou Kimura.

Writing edits:

1. Introduction: The Introduction needs to be expanded to place the work in a broader context and to appeal to a broader readership. As it is currently written, it is far too narrowly focused even for the C. elegans community.

2. Methods: Please provide additional details in the Methods regarding the 'hatch-off' protocol, the new microfluidics device, and the chemotaxis assay (please see note about chemotaxis assay below). Also, please provide relevant details regarding the parameters for the calcium imaging experiments (or include reference to previous work if parameters are the same), including the version of GCaMP used in each experiment.

3. Chemotaxis assay: Please specifically note duration time and scale for the assay in the figure or figure legend. It seems like the L3s are not as efficient at chemotaxing to nearly all odorants, and since this effect generalizes across attractants, it is possible that their small size and reduced crawling speed makes them less efficient at positive chemotaxis. The authors don't seem to have clearly distinguished size effects from age effects with their assay. Based on the Fujiwara paper, larvae do in fact have different odor preferences- Fujiwara et al. controlled for possible size effects by doing an odor preference assay and still saw differences in adults vs. larvae - but no similar control is provided here. Thus, while Fujiwara et al. demonstrate changes in odor preferences, it's less clear what the authors are looking at here. This point would be particularly interesting to address with regard to Figure 4, where inactivation of AWB, ASK, and AWC in adults reduces attraction. If an odor preference assay had been used for this experiment, and the AWB-, ASK-, AWC- adults showed reversed odor preferences that were analogous to the odor preferences of L3s, this would be more convincing. With only a regular chemotaxis assay, it's hard to know whether the reduced behavioral response is really analogous to an L3-like state.

4. The statement "transforms adults into a juvenile-like state" is a bit strong - please reword throughout.

5. Use of the term 'sensory circuit': Since you haven't shown that the sensory neurons you identified are technically in a circuit (for eg. not clear whether each neuron is a primary sensor or drives responses in a second neuron), please reword.

6. There are references missing throughout - TeTx, tom-1, the Chronis reference for the microfluidics device. Wrong reference in line 182 - Should be Chou, Bargmann and Sengupta 2001. Since the Fujiwara manuscript is now published, this work needs to be discussed and referenced.

7. Please check grammar and syntax. Reviewers noted multiple errors that I am not listing here. Please remove the last paragraph in the Discussion which is redundant.

8. Figure 3-2 is not included in the manuscript.

Statistics:

Please ensure that you have used the appropriate statistical test. In several cases shown, t-tests are not appropriate and data should be compared via ANOVA with a post-hoc correction for multiple comparisons (eg. Figs 1-1, 2. 2-1, 3, 3-1, etc).

Experimental:

1. Please show data for the responses of the triple-Tetx line (AWB ASK AWC) in the larval stage since the individual ablations have no effect in even the adult stage.

2. Can the tom-1 data be recapitulated with for instance pkc-1gf?

3. For Figure 5D, the n for the ASK-Tom1 is much smaller compared to the n shown for other data. A power analysis might be useful here to determine if you are underpowered.
